# *Neotrinia splendens* (*Liliopsida: Poaceae*) Growth Influences Spatial Distribution of Soil Bacterial Community in a Degraded Temperate Grassland

**DOI:** 10.3390/microorganisms13040894

**Published:** 2025-04-13

**Authors:** Jingjing Li, Qian Zhang, Yitong Chen, Mengmeng Diao, Chao Yang, Wenke Jia

**Affiliations:** 1 College of Grassland Science, Qingdao Agricultural University, Qingdao 266109, China; lijingjing@qau.edu.cn (J.L.); zq824316573@163.com (Q.Z.); mmengdiao@126.com (M.D.); 2 Shandong Key Laboratory for Germplasm Innovation of Saline-Alkaline Tolerant Grasses and Trees, Qingdao 266109, China; 3 College of Grassland Agriculture, Northwest A&F University, Yangling 712100, China; cyt990107@126.com

**Keywords:** grassland degradation, soil bacterial diversity, bacterial community composition, soil quality

## Abstract

*Neotrinia splendens* is widely distributed and is the dominant plant species of temperate degraded grassland in Inner Mongolia, showing a community growing habit forming a ring of individuals. However, there is a lack of attention to the soil microbial communities inside the ring (IN), outside the ring, and under the *N. splendens* ring (UN). This study investigated the soil bacterial community composition in three different zones of the *N. splendens* ring using amplicon sequencing technology, as well as soil environmental variables. The soil physicochemical properties, the composition of soil bacterial community, and the soil bacterial α-diversity varied significantly among the three zones. Especially, the growth of *N. splendens* promotes the soil bacterial diversity in the UN zone due to the interactions between plant and soil microbes. Soil NO_3_^−^-N, TC, TN, and pH are the key factors causing the variations of soil bacterial community composition and bacterial diversity. Proteobacteria and Actinobacteria phyla of microorganisms accounted for the largest proportion in network analysis among the three zones. Overall, attention should be paid not only to the improvement of grassland vegetation and soil quality but also to the change in soil microorganisms during the formation and expansion of the *N. splendens* ring in the future.

## 1. Introduction

Grasslands constitute the most extensive terrestrial environment in China, making up over 41% of the country’s total land area [1], and are primarily located in arid and semi-arid areas [2]. The grassland biome is challenged by physical disturbance and chemical pollution. However, due to the growing food demand, we need to increase the productivity of this ecosystem to support growing livestock production [3]. Furthermore, half of global grasslands are degraded to varying degrees [4], with 20% of these being severely degraded [5]. Grassland degradation is primarily caused by environmental and anthropogenic factors, including global warming, reduced precipitation, overgrazing, and random reclamation [6,7], which induced a significant decrease in grassland productivity and vegetation diversity. The secondary plant communities that are dominated by weeds supplant the primary plant communities [8]. Therefore, grassland ecosystems are facing serious degradation, which is manifested in various aspects, such as the decline in biodiversity, deterioration of environmental quality, reduced water retention capacity, decreased environmental productivity, and degradation of pasture quality [9].

Plant growth and productivity can be either positively or negatively impacted by the activity of a broad set of soil microbial communities [10]. Plants release a substantial portion (as much as 40%) of their photosynthetic products into the rhizosphere [11], known as root exudates, which orchestrate the dynamic assembly of the rhizosphere microbiome. Increasing evidence suggests that root exudates modulate the composition and structure of the rhizosphere microbiome by nourishing beneficial taxa, which in turn enhance the plant’s nutrient acquisition and immune response [12,13]. Soil microorganisms can drive material flow and energy transfer throughout environments, exerting important influences on the maintenance, development, and stability of grassland ecosystems [14]. These interactions also preserve nutrients needed for aboveground vegetation, influence the selection of plant traits, and influence plant diversity and productivity [10,15]. Grassland degradation is known to decrease the diversity of soil microbes and change the composition of their communities [16]. Furthermore, microbial communities exhibit a high degree of organization, forming intricate networks through mutual interactions or co-occurring groupings [17]. In light of *N. splendens’* wide distribution over China’s degraded grasslands, it is critical to investigate the changes in soil microbiota before and after the occurrence of the *N. splendens* ring and the relationships between soil microbes and physicochemical parameters in different zones of the *N. splendens* ring of degraded grassland.

*N. splendens* has a wide distribution [18] and is the dominant plant species of temperate degraded grassland in Inner Mongolia [19]. *N. splendens* grows in clones that increase area with increasing age, leading to different sizes of *N. splendens* that often generally exhibit ring patterns with a diameter of 20–150 cm [20]. Increased age leads to increased stem and leaf hardness, severe fibrosis, poor palatability, and difficulty in chewing and digesting by livestock [21]. The growth of *N. splendens* hinders the development of dominant herbaceous species in grassland, leading to a reduction in grassland biomass and degradation of the grassland ecological environment [22]. Hence, it is very important to understand the soil microbial changes and influencing mechanisms before and after the formation of the *N. splendens* ring of degraded grassland. We previously observed that the relative abundances of the primary bacteria in *N. splendens* rhizospheres significantly varied on a large regional scale [19]. Our previous study had focused on comparing the relationship between *N. splendens* rings of different diameters and their rhizosphere soil bacterial communities on a large scale. However, there is a lack of attention to the soil bacterial communities inside (IN), outside (OUT), and under the *N. splendens* ring (UN), that is, the variations in soil microbiota during and after the occurrence of the *N. splendens* ring of degraded grassland.

Our study has the following aims: (1) to investigate the soil physicochemical parameters in different zones of the *N. splendens* ring, (2) to characterize variation patterns of composition and diversity of soil bacteria in different zones of *N. splendens* ring, and (3) to analyse the relationship between soil physicochemical parameters and soil bacterial diversity and community composition in different zones of *N. splendens* ring of degraded grassland.

## 2. Materials and Methods

### 2.1. Study Site and Soil Collection

**Figure 1:**
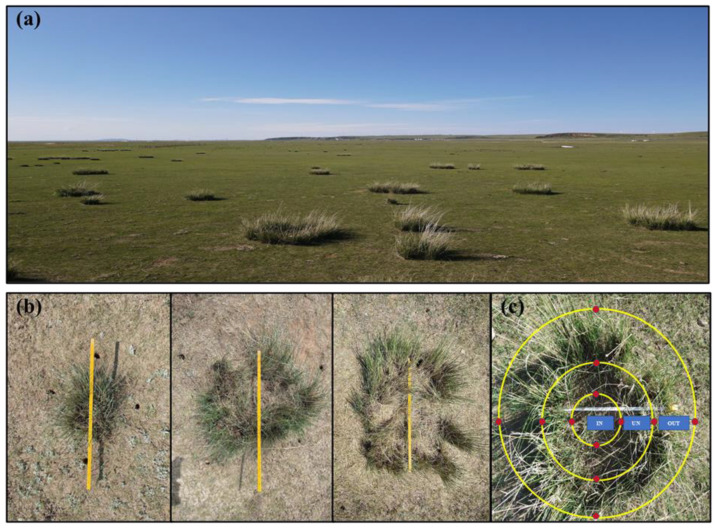
Distribution status of *N. splendens* in degraded grasslands of Inner Mongolia (**a**), and *N. splendens* ring with different sizes (**b**), and the division of three sampling zones (IN: inside the ring, UN: under the ring, and OUT: outside the ring), which the red points show the four sampling directions in each ring (**c**).

The study site was located at the temperate grassland (43°23′ N, 116°40′ E, altitude 1232 masl.) of Chifeng City in Inner Mongolia (Figure 1a). The average annual temperature and precipitation of this site are 1.5 °C and 250 mm, respectively. The dominant plant species are *Stipa grandis* (*Liliopsida: Poaceae*) and *Artemisia frigida* (*Magnoliopsida: Asteraceae*). The soil type is grassland chestnut soil. Soil samples were collected at the end of May 2022. Before sampling, taking the occurrence of *N. splendens* ring as a reference, we determined it as the UN zone, the inner ring as the IN zone, and the outer ring as the OUT zone. Thus, we divided the sampling area into three different zones. Composite soil samples (0–20 cm in depth) were systematically collected proximal to plant roots from four cardinal directions utilizing a 3 cm diameter soil auger. A total of 75 soil samples were collected (3 zones × 25 replicates) (Figure 1b,c). We divided each soil sample into two components, with one component brought back to the laboratory for air drying and determination of basic physicochemical parameters, and the other component was stored in the refrigerator at −80 °C for molecular-based community compositional analyses.

### 2.2. Determination of Soil Physicochemical Properties

Soils were allowed to air dry in the laboratory before being carefully screened using a 2-mm filter to ascertain the physicochemical parameters of the material. After 30 min of shaking a 1:2.5 air-dried soil/water solution, the soil pH and electrical conductivity (EC) were determined using a pH meter. The soil total carbon [23] and total nitrogen (TN) were assessed using an elementary analyzer (Vario EL cube, Frankfurt, Germany). Soil NH_4_^+^-N and NO_3_^−^-N concentrations were measured with a Continuous Flow Analytical System (AutoAnalyzer 3, Hamburg, Germany) after extracting with 2 M KCl [24].

### 2.3. Soil DNA Extraction and PCR Amplification

The microbial DNA in the soil was extracted using an E.Z.N.A.^®^ soil DNA kit (Omega Bio-tek, Norcross, GA, USA). The quality of the extracted DNA was assessed through 1% agarose gel electrophoresis and analysis of DNA concentrations and purity with a NanoDrop2000 spectrophotometer (Thermo Scientific, Waltham, MA, USA). The V3–V4 hypervariable regions of 16S rRNA genes were amplified from the DNA extracts using the forward and reverse primers 338F/806R, respectively [25], with barcode sequences attached to the primers. On an ABI Gene Amp^®^ 9700 type thermal cycler (Thermo Scientific, Waltham, MA, USA), the amplification process comprised pre-denaturation at 95 °C for 3 min, 27 cycles of denaturation at 95 °C for 30 s, annealing at 55 °C for 30 s, and extension at 72 °C for 30 s. A stable extension step was then performed at 72 °C for 10 min, and a final hold was performed at 4 °C. Every sample was subjected to three PCR duplicates. AxyPrep DNA Gel Extraction Kit (Axygen Biosciences, Union City, CA, USA) was used to purify the PCR products from the same sample, which were then pooled and recovered from 2% agarose gel electrophoresis. Amplicon concentrations were measured using a Quantus^TM^ Fluorometer (Promega, Madison, WI, USA).

### 2.4. Sequencing Library Construction

The NEXTFLEX Rapid DNA-Seq Kit (New England Biolabs, Ipswich, MA, USA) was utilized to produce a purified PCR product library. Initially, linkage sequences were linked, and magnetic bead screening was employed to eliminate connector self-connecting fragments. Subsequently, the library templates were enriched through PCR amplification, and magnetic beads were recovered from the PCR products to obtain the final library.

### 2.5. High-Throughput Sequencing Data Analysis

Paired-end raw sequences underwent quality filtering using the fastp software program [26] (https://github.com/OpenGene/fastp, version 0.19.6, accessed on 2 January 2019) and the FLASH program for merging [27] (https://ccb.jhu.edu/software/FLASH/, version 1.2.11). First, sequences were quality-filtered based on removal of sequences with quality scores less than 20 at the ends of reads, while considering a 50-bp window. If the average quality value in the window was less than 20, the bases at the end were removed from the window, and reads less than 50-bp were eliminated, as were those containing N bases. Second, the paired reads were merged into contiguous sequences based on overlap, with a minimum overlap length of 10 bp. Third, the maximum allowable mismatching ratios of overlapping regions of spliced sequences were set at 0.2, with sequences removed outside of this threshold. Fourth, samples were distinguished by barcode and primer sequences at both ends of the sequence, followed by adjustment of sequence direction. The allowed mismatch ratio for barcodes was set at 0.2 within the UPARSE [28] software program (http://drive5.com/uparse/, version 7.1). Remaining sequences after quality filtering were clustered into operational taxonomic units (OTUs) at the 97% nucleotide identity threshold, followed by removal of chimeras. To mitigate the potential impact of sequencing depth on subsequent α-diversity analyses, the number of sequences in all samples was subsampled to 20,000. This adjustment still allowed for an average good coverage value of 99.09% for each sample. OTUs were taxonomically annotated by comparison against the Silva 16S rRNA gene database (version 138) using the RDP classifier [29] (https://sourceforge.net/projects/rdp-classifier/, version 2.11) with a confidence threshold of 70%. The taxonomic composition of each community was evaluated at different species taxonomic levels. Functional prediction analysis was conducted from the 16S rRNA gene data using the PICRUSt2 (version 2.2.0) software program [30]. The raw sequencing data for bacteria were archived at the Sequence Read Archive (SRA) of the NCBI database under accession number SUB13860725.

### 2.6. Statistical Analysis

The QIIME2 software [31] (2019.4) was used to conduct rarefaction analysis of the OTU diversity, with a minimum level of depth set at 10, the lowest sequencing depth for all samples as 95%, and the selection of 10 depth values between two depths. Each community was rarified 10 times, followed by evaluation of the Chao1 richness, the number of observed species, and the Shannon diversity. The differences in soil physicochemical properties (pH, EC, NH_4_^+^-N, NO_3_^−^-N, TC, TN, and C/N ratio) and bacterial diversity indicators (Chao1 richness, observed species richness, and Shannon diversity) among the three zones were assessed using one-way ANOVA. Homogeneous groups of means were distinguished at *p* < 0.05 using Duncan’s tests in SPSS 19.0 (IBM, Armonk, NY, USA).

The soil bacterial ordination analysis among the three zones at the OTU level was determined using nonmetric multidimensional scaling (NMDS), and the significance of the results was assessed using permutational multivariate analysis of variance (PERMANOVA) in R statistical software [32] (ver. 3.6.3). The microeco package [33] of R statistical software (ver. 3.6.3) was used to conduct a random forest analysis to estimate the significance of the relative abundance of the bacterial community at the family level in the three zones.

The GIANT enhancement package of Cytoscape (ver. 3.9.1) [34] was used to analyze the Zi and Pi score. The module consists of a collection of OTUs that have strong relationships with one another but weaker relationships with OTUs in other modules [35]. Each OTU’s role was established by evaluating its standing in relation to other OTUs within its own module and the quality of its connections to nodes in other modules. Consequently, the inside-module connectivity (Zi) and among-module connectivity (Pi) of OTU i define its function within the network [36]. All species were divided into four subgroups based on the streamlined criteria: peripherals, connections, module hubs, and network hubs [35].

The SparCC algorithm [37] was used to construct a correlation matrix, and random matrix theory was used to determine the filtering threshold for the correlation value, followed by visualization of the associated network data in the igraph software (ver. 0.5.2) [38]. The induced subgraph function of igraph was then used to extract the nodes with the 100 highest average abundances to generate a dominant species subnetwork, followed by visualization with the graph package for R. Negative correlations within the network were removed to build a co-occurrence network. The “multi-level modularity optimization algorithm” of igraph was then used to modularly excise the co-occurrence network. The gml network file was also imported into the Gephi software (ver. 0.9.2) [39]. This study used a set of factors to characterize the topological features of networks, including diameters, average path lengths, and clustering coefficients. These parameters’ definitions can be found in Berry and Widder [40].

Redundancy analysis (RDA) was used to investigate the link between the soil bacterial community at the family level and soil physicochemical characteristics, such as pH, EC, NH_4_^+^-N, NO_3_^−^-N, TC, TN, and C/N ratio using R statistical software (ver. 3.6.3). We were able to ascertain the correlations between the soil factors and the distances separating microbial communities in PASSAGE (permutations = 999) by partial Mantel tests, and the Mantel r statistic and *p* values were used to examine the significance level that resulted. The association between bacterial relative abundance at the family level, bacterial diversity indices, and soil physicochemical parameters was depicted in a Pearson correlation heatmap, with the false discovery rate being controlled by an adjusted *p*-value.

## 3. Results

### 3.1. Changes in Soil Physicochemical Properties in Three Zones of the N. splendens Ring

The soil physicochemical properties in three zones of the *N. splendens* ring are shown in Table 1. Soil pH showed significant changes among the three zones, and the OUT zone was significantly higher than that in the IN and UN zones (*p* < 0.05). Soil NO_3_^−^-N and TC showed the same changes among the three zones, and significantly decreased from the IN zone to the OUT zone (*p* < 0.05). Soil NH_4_^+^-N of the OUT zone was significantly lower than that in the IN and UN zones (*p* < 0.05). In addition, TN in the IN zone was significantly higher than that in the UN and OUT zones (*p* < 0.05). Although TC showed significant differences among the three zones, the fluctuation of TN was relatively small. Thus, the fluctuation of C/N ratios was also relatively small, and only the IN zone was significantly higher than that in the OUT zone (*p* < 0.05).

### 3.2. Variations in Soil Bacterial Diversity and Community Composition in Three Zones of the N. splendens Ring

**Figure 2:**
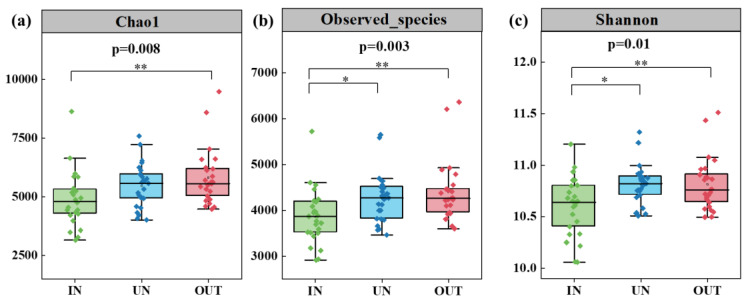
Soil bacterial Chao1 richness (**a**), observed species richness (**b**), and Shannon diversity (**c**) in three zones of the *N. splendens* ring (IN: inside the ring, UN: under the ring, and OUT: outside the ring). Asterisks indicate significant differences in three zones. * and ** indicate significant levels at *p* < 0.05 and *p* < 0.01, respectively.

**Figure 3:**
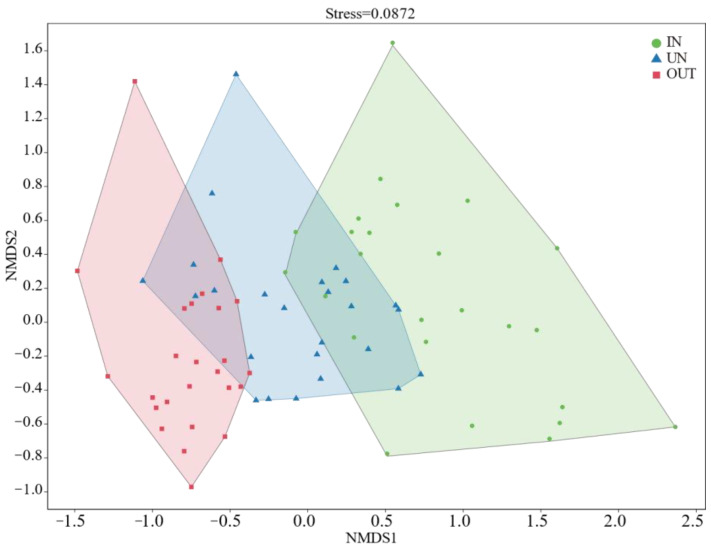
The non-metric multidimensional scaling (NMDS) ordinations based on the relative abundance of the bacterial communities at the OTU level in the three different zones of the *N. splendens* ring (IN: inside the ring, UN: under the ring, and OUT: outside the ring).

Compared with the IN zone, the Chao 1 index of bacteria in the OUT zone has significantly increased (Figure 2a, *p* = 0.008). In addition, the observed species index and the Shannon index showed the same tendency. These two indices in the UN zone and the OUT zone were significantly higher than those in the IN zone (Figure 2b,c, *p* < 0.05). The NMDS showed that the bacterial compositions in the IN zone differed from that in the OUT zone at the OTU level (Figure 3; stress = 0.0872), and PERMANOVA further confirmed that the Bray–Curtis distance between soil samples was greater than that within soil samples at the OTU level (*R* = 0.324, *p* = 0.001) (Table S1).

**Figure 4:**
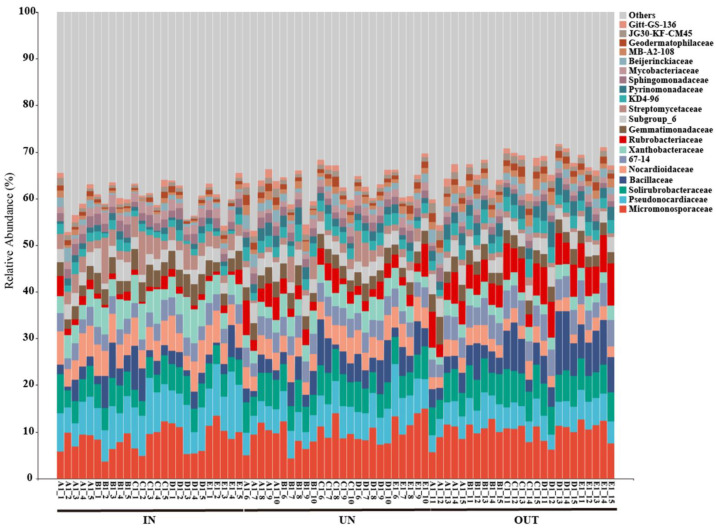
Relative abundance of the bacterial communities at the family level in three different zones of the *N. splendens* ring (IN: inside the ring, UN: under the ring, and OUT: outside the ring).

The relative abundance of the main dominant bacteria at the family level in three different zones was depicted in Figure 4. Overall, the Micromonosporaceae and Pseudonocardiaceae were the main microflora in all samples at the family level, accounting for 15.31% to 16.75% of total bacterial sequences among different zones, while JG30-KF-CM45 and Gitt-GS-136 were the minor microflora, accounting for 1.44% to 2.43% of all bacterial sequences (Figure 4). Specifically, the abundance of the Pseudonocardiaceae, Nocardioidaceae, Xanthobacteraceae, Streptomycetaceae, and Mycobacteriaceae was significantly decreased from the IN zone to the OUT zone. However, the abundance of the 67-14, Rubrobacteriaceae, Pyrinomonadaceae, Beijerinckiaceae, MB-A2-108, Geodermatophilaceae, and JG30-KF-CM45 were significantly increased from the IN zone to the OUT zone (Table S2).

Random forest analysis heat map displayed the top 20 most important indicator species that have an important impact on differences between the three zones. The result graph consists of two parts. The right side was a histogram of the importance score of each indicator species, which revealed that Rubrobacteriaceae, Devosiaceae, and Micropepsaceae were the top 3 indicator species that have an important impact on differences between three zones, with contribution degrees of 0.057, 0.035, and 0.026, respectively (Figure S1, Table S3).

### 3.3. Topological Function and Network Analysis of Soil Microorganisms in Three Zones of N. splendens Ring

The Zi-Pi diagram showed the topological role in microbial networks consisting of all soil samples. Proteobacteria represented the center of the network hub section for the entire microbial network. Actinobacteria accounted for three of the ten bacterial module hubs; the remaining three belonged to other taxa, such as Finnicutes, Chloroflexi, Acidobacteria, and Bacteroides (Figure S2).

**Figure 5:**
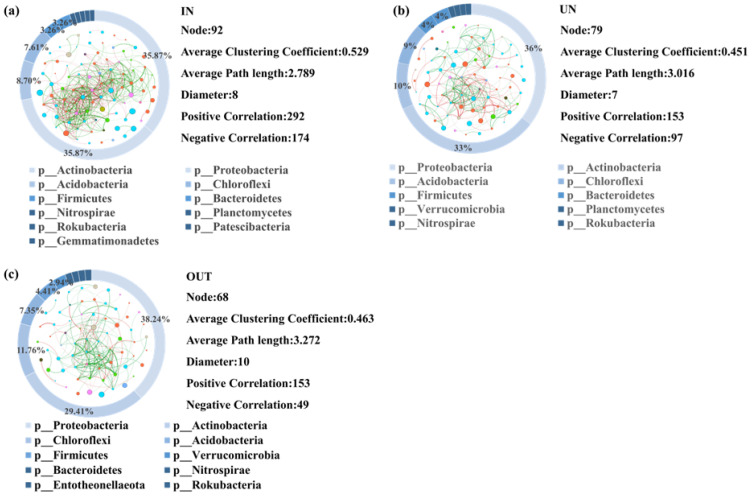
Network diagram showing the bacterial interactions in three zones of the *N. splendens* ring [IN: inside the ring (**a**), UN: under the ring (**b**), and OUT: outside the ring (**c**)], respectively. A red edge indicated a positive interaction between two individual nodes, while a green edge indicated a negative interaction. Each pie chart surrounding the network diagram represents the proportion of different bacteria in three zones of the *N. splendens* ring, which are represented by different colors.

A network was constructed to visualize all correlations between microorganisms of the three different zones (Figure 5). A total of 92 nodes between microorganisms were detected in the IN zone. Among them, there were 292 positive correlations and 174 negative correlations (Figure 5a). The Proteobacteria and Actinobacteria phyla of microorganisms accounted for the largest proportion in network analysis, with 35.87% relative abundance in the IN zone (Figure 5a). There were 79 nodes between microorganisms in the UN zone, of which 153 were positively correlated, whereas 97 were negatively correlated (Figure 5b). Proteobacteria and Actinobacteria phyla of microorganisms still occupied the largest proportion in network analysis, with 36% and 33% relative abundance in the UN zone, respectively (Figure 5b). Furthermore, a total of 68 microbial nodes were found in the OUT zone, of which 153 were positively correlated and 49 were negatively correlated (Figure 5c). Similar to the IN and UN zones, Proteobacteria and Actinobacteria phyla of microorganisms had the greatest contribution in network analysis, with 38.24% and 29.41% relative abundance in the OUT zone, respectively (Figure 5c).

### 3.4. Relationships Between Soil Physicochemical Properties and Soil Bacterial Diversity and Community Composition

The RDA biplots indicated that a specific set of variables explained 33.45% of the observed variation in bacterial communities at the OTU level across the three zones (Figure S3). Mantel tests revealed a significant association between the bacterial communities at the OTU level and the soil physicochemical properties, including soil NO_3_^−^-N, NH_4_^+^-N, TC, TN, pH, and C/N (Table S4).

**Figure 6:**
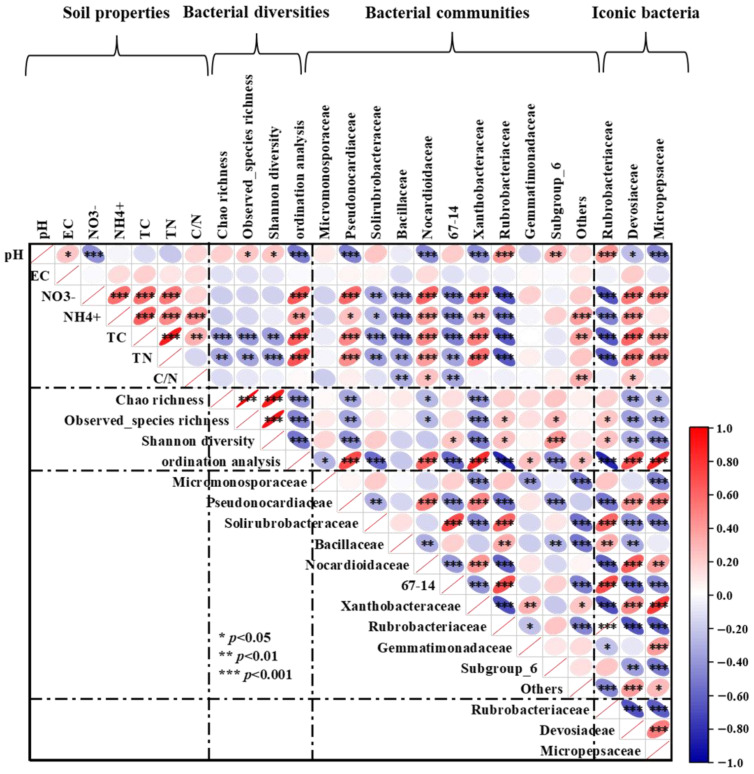
Pearson correlation heatmap shows the relationship between soil physicochemical properties, bacterial classification at the family level, bacterial diversity indices, and iconic bacteria. EC: soil electrical conductivity; TC: soil total carbon; TN, soil total nitrogen; and C/N ratio: soil carbon/nitrogen ratio. Red ellipses sloping up indicate a positive correlation, while blue ellipses sloping down indicate a negative correlation. The width of the ellipse indicates the level of correlation. *, **, and *** indicates statistical significance of the path at the *p* < 0.05, *p* < 0.01, and *p* < 0.001 levels, respectively.

Soil pH was found to be considerably and favorably correlated with observed species richness and Shannon diversity, according to Pearson correlation analysis. Soil TC and TN had a significantly adverse correlation with bacterial α-diversity, including Chao 1 richness, observed species richness, and Shannon diversity. The soil pH exhibited a strong negative correlation with the relative abundance of Pseudonocardiaceac, Nocardioidaceae, Xanthobacteraceae, and Devosiaceae, but a strong positive correlation with Rubrobacteriaceae. On the contrary, soil NO_3_^−^-N, NH_4_^+^-N, TC, and TN were considerably and favorably correlated with the relative abundances of Pseudonocardiaceac, Nocardioidaceae, Xanthobacteraceae, and Devosiaceae, but significantly and adversely correlated with Rubrobacteriaceae. In addition, soil NO_3_^−^-N, NH_4_^+^-N, TC, and TN showed a strong negative correlation with the abundance of Solirubrobacteraceae, Bacillaceae, and 67-14. Soil C/N ratio was positively correlated with the relative abundances of Nocardioidaceae and Devosiaceae, but negatively correlated with Bacillaceae, and 67-14 (Figure 6).

## 4. Discussion

### 4.1. Soil Physicochemical Properties in Different Zones of N. splendens Ring

Soil physicochemical properties are crucial for assessing soil health and establishing the basis for biological activity in the soil [41]. In the present study, all of the soil observation indicators (i.e., pH, NO_3_^−^-N, NH_4_^+^-N, TC, TN, and C/N), except EC, showed significant variation among three zones of the *N. splendens* ring. This indicated that the formation and expansion of the *N. splendens* ring would change the physicochemical properties of the soil in which it is located. This may be due to the correlation between plants, root exudates, and soil microorganisms that causes changes in soil properties [11]. The pH level of soil plays a crucial role in regulating various aspects of soil fertility and biogeochemical cycling [42]. Soil pH in the OUT zone was significantly higher than that in the other two zones due to the fact that the research area belongs to soda saline alkali land. Soil nutrients originate from the soil parent material, plant exudates, litter, and animal excreta [43,44]. Our study showed that the formation and spread process of the *N. splendens* ring reduced nutrient levels such as soil NO_3_^−^-N, NH_4_^+^-N, TC, and TN, which showed a decreasing trend from the IN zone to the OUT zone.

### 4.2. Characteristics of Soil Microbial Community in Different Zones of N. splendens Ring

The diversity of the microbial community is commonly utilized as an indicator of the dynamic changes occurring in soil microbes during ecological processes [45]. According to Figure 2, the differences in bacterial diversity of the three zones could be clearly seen. Chao 1 index, observed species index, and the Shannon index showed an increasing tendency from the IN zone to the OUT zone. Soil bacterial diversity is significantly associated with plant growth [46]. In our study, the observed species index and the Shannon index in the UN zone and the OUT zone were significantly higher than those in the IN zone (*p* < 0.05), which indicated that the growth of *N. splendens* promotes the diversity of soil microorganisms in the UN zone. That is, following the expansion of the *N. splendens* ring, the diversity of soil bacteria inside the ring has decreased. In terrestrial ecosystems, primary productivity and ecosystem functioning are determined by the interactions and feedbacks between soil microbes and plants [17,47]. Plant roots nearby have the ability to modify microbiological processes within soil. Since mucilage and root exudates are major sources of energy for many bacteria, plant roots emit organic chemicals that alter microbial catabolic activity and functional diversity [48]. This implied that the formation and spread process of the *N. splendens* ring had changed the soil bacterial diversity, especially increased soil bacterial diversity in the UN zone, which might be due to the root system of *N. splendens* providing a food source for the microorganisms.

In terrestrial ecosystems, soil microbes play a critical role in biological diversity and ecosystem processes [49]. The microbial community in soil is typically stable. However, the emergence and growth of the *N. splendens* ring will upset the ecological balance and push the original microorganisms to adjust to their new surroundings, changing the composition and variety of the microbial community [50]. In the present study, the formation and expansion of the *N. splendens* ring significantly affected the soil bacterial community composition. In the analysis of the relative abundance of bacterial communities, we found that different microbial groups exhibited different response patterns to the expansion of the *N. splendens* ring. The abundance of the Pseudonocardiaceae, Nocardioidaceae, Xanthobacteraceae, Streptomycetaceae, and Mycobacteriaceae was significantly decreased from the IN zone to the OUT zone (*p* < 0.05). However, some other microbial groups (i.e., 67-14, Rubrobacteriaceae, Pyrinomonadaceae, Beijerinckiaceae, MB-A2-108, Geodermatophilaceae, and JG30-KF-CM45) showed opposite patterns of change, which were significantly increased from the IN zone to the OUT zone (*p* < 0.05). This result can be inferred from the formation and expansion of the *N. splendens* ring, which can be evidenced through the variation of the microbial community structure. Furthermore, Proteobacteria played a pivotal role in bacterial networks, while the phylum Acidobacteria and Chloroflexi served as module-hubs or connectors within the networks. Proteobacteria and Actinobacteria phyla of microorganisms accounted for the largest proportion in network analysis among the three zones, indicating a potential conservation of functional roles at the phylum level of the bacterial community.

### 4.3. Relationship Between Soil Physicochemical Properties and Microorganisms

Soil microorganisms play a significant role in regulating the physicochemical qualities of the soil by altering the diversity and composition of the soil microbial communities [51]. Soil microbial communities were driven by the intimate relationship between soil physicochemical properties and microorganisms within the ecosystem [52]. In this study, we found that the matrix of soil physicochemical properties had a significant impact on bacterial compositions as determined by the partial Mantel test. This was observed for most individual physicochemical factors. It was suggested that soil nutrients, particularly the effectiveness of C and N, were the primary influence shaping microbial composition [53], which was consistent with our study. Highly significant correlations between the contents of soil NO_3_^−^-N, NH_4_^+^-N, TC, TN, C/N, and microbial compositions were determined using Mantel tests. Additionally, the Pearson correlation analysis also showed that soil NO_3_^−^-N, NH_4_^+^-N, TC, and TN had a significant positive correlation with the relative abundances of Pseudonocardiaceac, Nocardioidaceae, Xanthobacteraceae, and Devosiaceae, but a significant negative correlation with the primary bacteria including Solirubrobacteraceae, Bacillaceae, Rubrobacteriaceae, and 67-14. The soil C/N ratio had a positive correlation with the relative abundances of Nocardioidaceae and Devosiaceae, but a significant negative correlation with Bacillaceae and 67-14. The impact of pH on soil microbial distribution patterns has been widely documented in numerous studies [54,55]. The study also revealed that soil microbial community composition was significantly influenced by pH. However, different microbial populations exhibited varied responses to pH; for instance, the abundances of Pseudonocardiaceac, Nocardioidaceae, Xanthobacteriaceae, and Devosiaceae showed a negative correlation with pH, whereas the abundances of Rubrobacteriaceae exhibited a positive correlation with pH.

A variety of soil physicochemical parameters also influence bacterial diversity, which is in line with previous research findings [56]. Studies have indicated that an increase in pH value leads to higher bacterial diversity and microbial activity, while a decrease in soil pH results in lower bacterial and microbial activity. Therefore, pH serves as a reliable predictor of bacterial community composition [57,58]. Here, we found that soil pH was significantly and positively correlated with α-diversity, including observed species richness and Shannon diversity. It was reported that soil bacterial α-diversity is positively correlated with pH in the range of 4–8, but negatively correlated with pH when pH > 8 [59]. This was consistent with our findings. The soil pH of the three zones ranged from 6.98 to 7.45, thus showing a positive correlation between pH and soil bacterial α-diversity in our study. Multiple studies have reported a negative association between soil bacterial diversity and total C, suggesting that soil degradation and drought result in reduced soil organic carbon, leading to an increase in bacterial diversity [60,61,62], especially in arid soils [63]. This was consistent with our findings. Soil TC had a significant negative correlation with bacterial α-diversity, including Chao 1 richness, observed species richness, and Shannon diversity. Ordination analysis showed that different zones had a strong influence on the soil microbial community. In other words, the formation and expansion of the *N. splendens* ring may lead to significant differences in the structure of microbial communities in soil.

## 5. Conclusions

This study investigated the dynamics of soil bacterial communities during the formation and expansion of *N. splendens* rings in a temperate grassland ecosystem in Inner Mongolia. Significant variations in soil physicochemical properties—except for electrical conductivity (EC)—were observed across the three distinct zones. At the family level, the relative abundance of dominant bacterial taxa exhibited notable differences, with Micromonosporaceae and Pseudonocardiaceae emerging as the predominant microflora. Following the expansion of the *N. splendens* ring, the diversity of soil bacteria inside the ring has decreased. This suggests that for the sake of maintaining soil health and preserving microbial diversity, we should prioritize prevention and control measures before the *N. splendens* ring expands further.

## Figures and Tables

**Table 1:** Root-associated soil physicochemical properties of the *N. splendens* ring based on different zones (IN: inside the ring, UN: under the ring, and OUT: outside the ring). Values are Mean ± standard error.

Three Zones	pH	EC (μs cm^−1^)	NO_3_^−^-N (mg kg^−1^)	NH_4_^+^-N (mg kg^−1^)	TC (g kg^−1^)	TN (g kg^−1^)	C/N Ratio
IN	6.98 ± 0.09 A	1004.33 ± 44.91 A	35.37 ± 1.19 C	13.31 ± 0.66 B	1.95 ± 0.08 C	0.15 ± 0.01 B	12.67 ± 0.55 B
UN	7.20 ± 0.08 B	988.22 ± 29.84 A	31.25 ± 1.03 B	11.77 ± 0.71 B	1.31 ± 0.04 B	0.11 ± 0.00 A	12.02 ± 0.43 AB
OUT	7.45 ± 0.05 C	972.19 ± 43.61 A	15.89 ± 0.86 A	6.70 ± 0.30 A	1.04 ± 0.04 A	0.09 ± 0.00 A	11.06 ± 0.24 A

Note: EC: soil electrical conductivity; TC: soil total carbon; TN: soil total nitrogen; C/N ratio: soil carbon/nitrogen ratio. Different capital letters indicate differences between the three zones (*p* < 0.05).

## Data Availability

The original data generated in this study are included in this article. Further enquiries can be directed to the corresponding authors.

## Supplementary Materials

The following supporting information can be downloaded at: https://www.mdpi.com/article/10.3390/microorganisms13040894/s1, Table S1: PERMANOVA analysis between soil samples at the OTU level in three different zones of the *N. splendens* ring (IN: inside the ring, UN: under the ring, and OUT: outside the ring); Table S2: Relative abundance of soil bacteria at the family level in three zones of the *N. splendens* ring (IN: inside the ring, UN: under the ring, and OUT: outside the ring). Values are Mean ± SE. Lowercase letters indicate difference between three zones (*p* < 0.05); Table S3: The contribution degrees of the top 20 most important indicator species based on the random forest analysis of the *N. splendens* ring; Table S4: Partial Mantel tests between the soil properties and the bacterial community structures at the OTU level of the *N. splendens* ring; Figure S1: Random forest analysis heat map displayed the top 20 most important indicator species that have an important impact on differences between three zones (IN: inside the ring, UN: under the ring, and OUT: outside the ring). The right side was a histogram of the importance score of each indicator species; Figure S2: Zi-Pi plot showing the distribution of OTUs based on their topological roles in networks between bacteria. Each circle of different colors represented an OTU in the bacterial network; Figure S3: Redundant analysis showing the effects of soil physicochemical properties (pH, EC, NO_3_^−^-N, NH_4_^+^-N, TC, TN, and C/N) on bacterial community structure at the family level in three different zones of the *N. splendens* ring (IN: inside the ring, UN: under the ring, and OUT: outside the ring). Partial Mantel tests (permutation = 999) were used to assess the statistical significance of the effects of each property. EC: soil electrical conductivity; TC: soil total carbon; TN: soil total nitrogen; C/N: soil carbon/nitrogen ratio.
