# Supplementary material for: Neotrinia splendens (Liliopsida: Poaceae) Growth Influences Spatial Distribution of Soil Bacterial Community in a Degraded Temperate Grassland
Source: Microorganisms. 2025 Apr 13;13(4):894. doi: 10.3390/microorganisms13040894 (PMC12029622; doi:10.3390/microorganisms13040894)
Supplement: Supplementary file 1 [file microorganisms-13-00894-s001.zip › microorganisms-3548713-supplementary.pdf]

Supplementary Material

Neotrinia Splendens (*Liliopsida: Poaceae*) Growth Influences Spatial Distribution of Soil Bacterial Community in a Degraded Temperate Grassland

Jingjing Li [1,2,†], Qian Zhang [1,2,†], Yitong Chen [3], Mengmeng Diao [1,2], Chao Yang [1,2,*] and Wenke Jia [1,2,*]

[1] College of Grassland Science, Qingdao Agricultural University, Qingdao 266109, China;
lijingjing@qau.edu.cn (J.L.); zq824316573@163.com (Q.Z.); mmengdiao@126.com (M.D.)
[2] Shandong Key Laboratory for Germplasm Innovation of Saline-Alkaline Tolerant Grasses and Trees, Qingdao 266109, China
[3] College of Grassland Agriculture, Northwest A&F University, Yangling 712100, China; cyt990107@126.com
* Correspondence: yangchao@qau.edu.cn (C.Y.); wenkejia@qau.edu.cn (W.J.)
† These authors contributed equally to this work.

Table S1. PERMANOVA analysis between soil samples at the OTU level in three different zones of the *N. splendens* ring (IN: inside the ring, UN: under the ring, and OUT: outside the ring).

Group1	Group2	Sample# size	Permutations	R	p# value	q# value
all	-	75	999	0.324485	0.001	-
IN	UN	50	999	0.220549	0.001	0.001
IN	OUT	50	999	0.534456	0.001	0.001
UN	OUT	50	999	0.228189	0.001	0.001

Table S2. Relative abundance of soil bacteria at the family level in three zones of the *N. splendens* ring (IN: inside the ring, UN: under the ring, and OUT: outside the ring). Values are Mean ± SE. Lowercase letters indicate difference between three zones ($P < 0.05$).

Bacterial classification	Relative abundance in three zones		
	IN（%）	UN（%）	OUT（%）
Micromonosporaceae	8.52±0.52a	9.71±0.55ab	10.13±0.38b
Pseudonocardiaceae	8.23±0.44c	6.24±0.3b	5.18±0.18a
Solirubrobacteraceae	5.07±0.23a	5.78±0.18b	6.32±0.17b
Bacillaceae	3.43±0.47a	4.49±0.46a	6.29±0.62b
Nocardioidaceae	5.48±0.25c	4.4±0.16b	3.08±0.13a
67-14	3.49±0.13a	3.92±0.1b	4.72±0.16c
Xanthobacteraceae	4.97±0.32c	3.76±0.2b	2.7±0.11a
Rubrobacteriaceae	1.4±0.18a	3.18±0.32b	5.86±0.34c
Gemmatimonadaceae	3.41±0.17b	2.97±0.11a	3.01±0.15ab
Subgroup_6	2.87±0.2a	3.14±0.14a	3.15±0.11a
Streptomycetaceae	3.57±0.41c	2.24±0.31b	1.05±0.06a
KD4-96	1.61±0.11a	2.41±0.09b	2.64±0.11b
Pyrinomonadaceae	1.02±0.11a	1.78±0.13b	2.68±0.16c
Sphingomonadaceae	1.91±0.08b	1.78±0.06b	1.33±0.08a
Mycobacteriaceae	1.85±0.08c	1.58±0.08b	1.12±0.07a
Beijerinckiaceae	0.89±0.12a	1.36±0.15b	1.78±0.1c
MB-A2-108	0.63±0.07a	1.3±0.08b	1.57±0.07c
Geodermatophilaceae	0.68±0.07a	1.02±0.11b	1.34±0.07c
JG30-KF-CM45	0.72±0.06a	0.93±0.07b	1.32±0.08c
Gitt-GS-136a	0.72±0.08a	1.06±0.08b	1.11±0.08b
other	39.59±0.68c	36.95±0.81b	33.62±0.82a

Table S3. The contribution degrees of the top 20 most important indicator species based on the random forest analysis of the *N. splendens* ring.

Bacterial classification	Contribution
Rubrobacteriaceae	0.057
Devosiaceae	0.035
Micropepsaceae	0.026
MB-A2-108	0.02
Xanthomonadaceae	0.0196
Pyrinomonadaceae	0.0196
Archangiaceae	0.0196
Beijerinckiaceae	0.0188
Thermomonosporaceae	0.0186
Inquilinaceae	0.0181
Roseiflexaceae	0.0175
Gaiellaceae	0.0171
Micrococcaceae	0.0169
Sphingobacteriaceae	0.0166
KD4-96	0.0163
Streptomycetaceae	0.0153
Reyranellaceae	0.0139
JG30-KF-CM45	0.0137
Latescibacteria	0.0133
Chloroflexaceae	0.0125

Table S4. Partial Mantel tests between the soil properties and the bacterial community structures at the OTU level of the *N. splendens* ring.

Partial Mantel tests	r^2	*p*-value
pH	0.3702	0.001
EC (μs cm^{-1})	0.0213	0.478
NO_3^--N(mg kg^{-1})	0.5667	0.001
NH_4^+-N(mg kg^{-1})	0.3331	0.001
TC (g kg^{-1})	0.5200	0.001
TN (g kg^{-1})	0.4262	0.001
C/N	0.1365	0.005

EC: soil electrical conductivity; TC: soil total carbon; TN: soil total nitrogen; C/N ratio: soil carbon/nitrogen ratio.

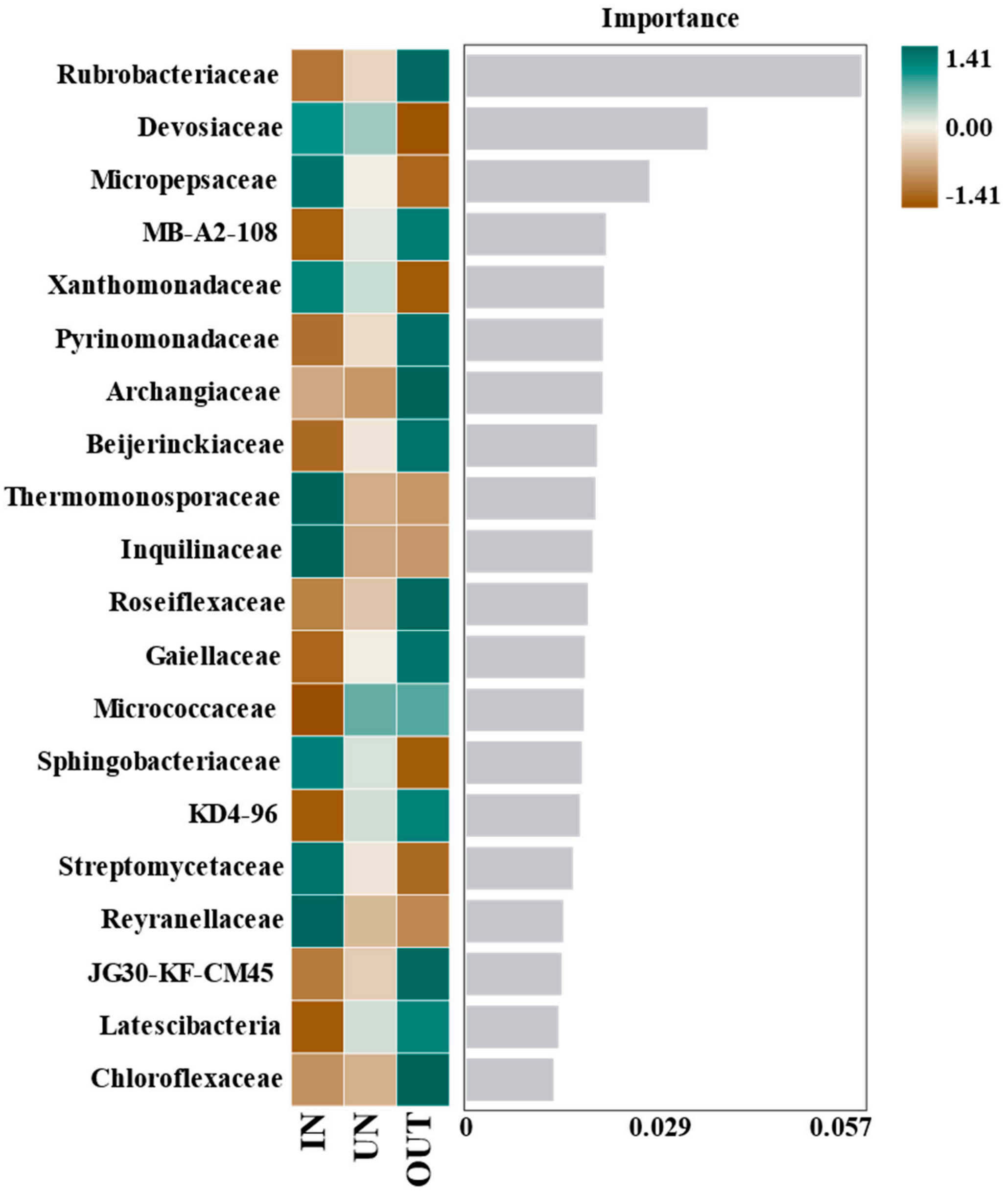

Figure S1. Random forest analysis heat map displayed the top 20 most important indicator species that have an important impact on differences between three zones (IN: inside the ring, UN: under the ring, and OUT: outside the ring). The right side was a histogram of the importance score of each indicator species.

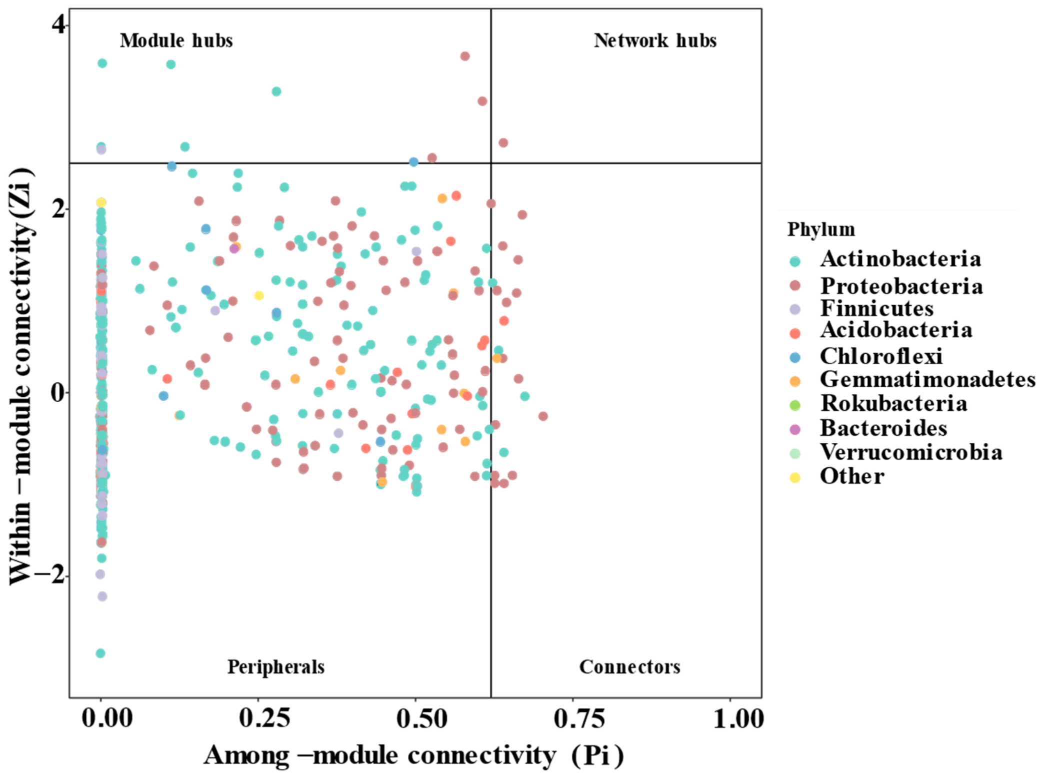

Figure S2. Zi-Pi plot showing the distribution of OTUs based on their topological roles in networks between bacteria. Each circle of different colors represented an OTU in the bacterial network.

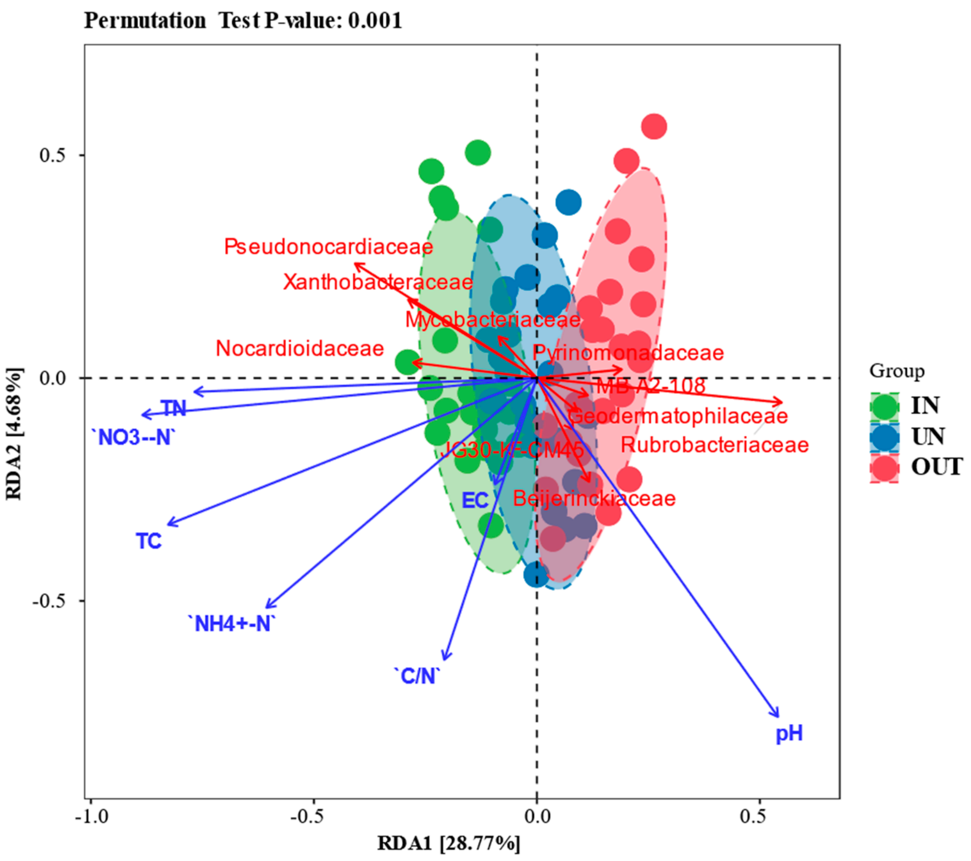

Figure S3. Redundant analysis showing the effects of soil physicochemical properties (pH, EC, NO_3^--N, NH_4^+-N, TC, TN, and C/N) on bacterial community structure at the family level in three different zones of the *N. splendens* ring (IN: inside the ring, UN: under the ring, and OUT: outside the ring). Partial Mantel tests (permutation = 999) were used to assess the statistical significance of the effects of each property. EC: soil electrical conductivity; TC: soil total carbon; TN: soil total nitrogen; C/N: soil carbon/nitrogen ratio.
